# The Association Between Proton Pump Inhibitors and COVID-19 is Confounded by Hyperglycemia in a Population-Based Study

**DOI:** 10.3389/fphar.2022.791074

**Published:** 2022-02-04

**Authors:** Asher Shafrir, Ariel A. Benson, Lior H. Katz, Tiberiu Hershcovici, Menachem Bitan, Ora Paltiel, Ronit Calderon-Margalit, Rifaat Safadi, Michal Shauly-Aharonov

**Affiliations:** ^1^ Faculty of Medicine, Hebrew University of Jerusalem, Jerusalem, Israel; ^2^ Meuhedet Health Medical Organization, Jerusalem, Israel; ^3^ Institute of Gastroenterology and Hepatology, Hadassah University Medical Center, Jerusalem, Israel; ^4^ Braun School of Public Health and Community Medicine, Hebrew University of Jerusalem, Jerusalem, Israel; ^5^ Department of Hematology, Hadassah University Medical Center, Jerusalem, Israel; ^6^ The Jerusalem College of Technology, Jerusalem, Israel

**Keywords:** COVID - 19, SARS-CoV-2, hyperglycaemia, proton pump inhibitor, impaired fasting blood glucose

## Abstract

**Background and Aims:** There is conflicting evidence regarding the association between proton pump inhibitors (PPI) and the risk of acquisition and severity of acute respiratory syndrome coronavirus 2 (SARS-CoV-2) infection.

**Aim:** To evaluate the association between PPI exposure and infection and development of severe disease in patients infected with SARS-CoV2in a large population-based historical cohort.

**Methods:** Data were extracted from a health maintenance organization database in Israel that insures over 1,200,000 individuals from across the country. All patients who underwent SARS-CoV-2 testing between March and November 2020 were included. Logistic regression and matched analyses were used to compare patients prescribed and exposed to PPIs to those not prescribed PPIs regarding SARS-CoV-2 positivity. In addition, among SARS-CoV-2 positive patients (n = 44,397) the likelihood of developing severe disease, defined by a composite endpoint of death, ICU admission and prolonged hospitalization, was compared in those exposed and not exposed to PPIs.

**Results:** Among 255,355 adult patients who underwent SARS-CoV-2 testing by PCR, 44,397 (17.4%) were positive for SARS-CoV-2 and 12,066 (4.7%) patients were prescribed PPIs in the 3 months before testing. In a multivariable logistic regression model controlling for age, gender, smoking status, BMI, diabetes mellitus, hypertension, COPD, history of ischemic heart disease and fasting blood glucose (FBG) levels, no significant association was found between PPIs and SARS-CoV-2 positivity (*p* = 0.09 aOR 0.94, 95% CI – 0.88–1.01). Among SARS-CoV-2 positive patients, 910 (2%) had a severe infection. Multivariate logistic regression controlling for the abovementioned confounders, showed no such association between PPIs and severe COVID-19 (*p* = 0.28). Elevated FBG levels were significantly associated with both PPI exposure (*p* < 0.001) and severe COVID-19 infection (*p* < 0.001). These results were reinforced by a matched analysis (n = 655 pairs).

**Conclusion:** PPIs are spuriously associated with severe COVID-19 due to the presence of elevated FBG as a confounder. Our study accounted for the FBG levels of patients and known risk factors for severe COVID-19 infection, which may be the reason for the discrepancy in prior studies. These results may aid in understanding potential confounders when evaluating potential associations of PPIs with other respiratory or viral diseases.

## Introduction

The COVID-19 pandemic caused by the severe acute respiratory syndrome coronavirus 2 (SARS-CoV-2) has led to worldwide morbidity and mortality (Grasselli et al., 2020). Several risk factors have been associated with worse outcomes among patients infected with SARS-CoV-2, including advanced age, obesity, and diabetes mellitus (DM) (Grasselli et al., 2020). In addition, studies have attempted to evaluate the association of several medications with both the risk of increased susceptibility to SARS-CoV-2 and the risk of more severe outcomes once infected (Fang et al., 2020). (Meng and Liang, 2021). Proton pump inhibitors (PPIs) are used extensively worldwide and can be purchased over the counter depending on the geographic location (Forgacs and Loganayagam, 2008; Katz et al., 2013). Numerous studies have proposed associations between PPIs and various adverse events (Dharmarajan, 2021). While many of these associations were not confirmed via randomized controlled trials (RCT), a RCT and a meta-analysis only confirmed the association between PPIs and enteric infections (D'Silva et al., 2021; Moayyedi et al., 2019).

PPIs inhibit hydrogen-potassium adenosine triphosphatase in gastric parietal cells, thereby decreasing gastric acidity (Engevik et al., 2020). Decreased gastric acidity is thought to cause an increased risk of enteric infections (Ray et al., 2020). At the same time, PPIs also may exert an anti-inflammatory effect, which may potentially be protective against SARS-CoV-2 (Ray et al., 2020; Conrad, 2021). Due to the widespread use of PPIs, their potential impact on SARS-CoV-2 should be carefully examined.

Studies evaluating the association between PPIs and SARS-CoV-2 positivity and COVID-19 disease severity based on self-reported patient surveys and health insurance claims databases showed conflicting results (Almario et al., 2020; Lee et al., 2020). PPIs were associated with more severe COVID-19 outcomes in a nationwide cohort from Korea, but there was no association between PPIs and SARS-CoV-2 positivity (Lee et al., 2020). A post-hoc analysis of this study showed a dose-dependent relationship between higher doses of PPIs and severe COVID-19 (Lee et al., 2021). A national health survey in the United States showed increased self-reported SARS-CoV-2 positivity among those who self-reported PPI use (Almario et al., 2020). A meta-analysis comprising highly heterogeneous studies showed a nonsignificant increased risk of SARS-CoV-2 but also a statistically significant increased risk of more severe COVID-19 outcomes among PPI users (Li et al., 2020). Another meta-analysis showed an increased risk of severe COVID-19 and the development of secondary infection in patients with prior PPI use (Kow and Hasan, 2021). A nationwide study from Denmark showed that current PPI use was associated with a moderately increased odds ratio for SARS-CoV-2 infection of 1.08 (95% CI, 1.03–1.13). Among SARS-CoV-2 cases, PPI use was associated with an increased risk of hospital admission but not with other severe COVID-19 related outcomes (Israelsen et al., 2013). On the other hand, a study of the UK Biobank found no increased risk of SARS-CoV-2 infection or death among PPI users, and an accompanying meta-analysis found no increased SARS-CoV-2 susceptibility with the use of PPIs or H-2 receptor blockers (Fan et al., 2021). Another large study from the UK Biobank, showed no association between PPI use and COVID-19-related mortality after adjusting for overall health status (He et al., 1430). These conflicting results call for additional well-designed studies to clarify the association of PPIs and COVID-19 severity (Lv et al., 2021).

Multiple risk factors for severe COVID-19 have been described, such as advanced age, male gender, cardiovascular disease, increased BMI, and respiratory disease (McGurnaghan et al., 2021; Wolff et al., 2021). A significant confounder when assessing SARS-CoV-2 positivity and COVID-19 severity is abnormal glucose levels in addition to diabetes mellitus (DM) (Zhang et al., 2020; Zhu et al., 2020). Recently, data from our health care maintenance organization (HMO) showed that impaired fasting glucose and dysglycemia among patients with diabetes (hypo or hyperglycemia in FBG tests) are associated with an increased risk for severe COVID-19 (Shauly-Aharonov et al., 2021). Prospectively collected data from a comprehensive electronic medical record that includes specific laboratory values and documentation of diagnoses and past medical history enables the assessment of the association between medication exposure and SARS-Cov2 infection and severity while evaluating the role of potential confounding factors.

## Methods

Meuhedet HMO is one of the four HMOs in Israel. It is Israel’s third-largest healthcare provider, serving over 1,200,000 individuals. Meuhedet’s computerized database includes real-time input from all physician visits, medical diagnoses, laboratory results, hospitalizations (including SARS-CoV-2 testing from all locations), and dispensing data on prescription and over-the-counter medications. Health data from the electronic medical records (EMR) of all insured individuals aged 18 and above who underwent a SARS-COV-2 polymerase chain reaction (PCR) test from 1 March 2020 to 30 November 2020, were extracted. Laboratory confirmation of SARS-COV-2 was defined as a positive result of a real-time PCR assay from nasal and pharyngeal swabs according to World Health Organization guidelines (World Health, 2020). If patients underwent multiple tests during this period, the first positive test was considered the index test. If all tests were negative, the first SARS-CoV-2 test was considered the index test.

Three sectors of Israeli society can be loosely identified based on the clinic’s location. These are the Arab, ultra-orthodox Jewish, and orthodox/secular Jewish. Recent studies showed differences in SARS-CoV-2 prevalence between these groups, and therefore, this variable was included as a potential risk factor (Dagan et al., 2021). Additional data extracted from patient EMRs included age, gender, body mass index (BMI), and significant medical diagnoses documented at any point before SARS-CoV-2 testing [e.g., ischemic heart disease (IHD), hypertension, DM, hyperlipidemia, hypothyroidism, chronic obstructive pulmonary disease (COPD)]. Prescriptions for omeprazole, esomeprazole, lansoprazole, and pantoprazole prescribed to patients in the 3 months before SARS-CoV-2 index test were also retrieved. In addition, we included results of the most recent FBG tests and glycosylated hemoglobin (HbA1C) performed within 1–12 months prior to SARS-CoV-2 testing.

COVID-19 outcomes were recorded, and the severity of outcomes was assessed. Severe infection was defined as any one of the following events: (1) death, (2) intensive care unit (ICU) admission, or (3) hospitalization of 10 days or more following the test.

This research was conducted in accordance with the Declaration of Helsinki and approved by the research ethics committee and internal review board of Meuhedet HMO (02-24-08-20).

## Statistical Analysis

For descriptive analysis, we used counts and percentages for categorical variables. Continuous variables were summarized as means and standard deviations (SD). Bivariate analysis was performed using the Chi-squared test to compare categorical variables and t-test to compare means of continuous variables. Logistic regression was performed to evaluate the association between PPIs and SARS-CoV-2 positivity and the association between PPIs and severe COVID-19 infection. Odds ratios (OR) and corresponding 95% confidence intervals (CI) were reported. A multivariate logistic regression model was constructed to control for confounders previously found to be associated with increased risk for severe COVID-19. To further assess the associations between PPIs and SARS-CoV-2, 1:1 matching was performed to equate the distribution of covariates in the treated (i.e., PPI users) and control group. Two closeness measures - propensity score (Rosenbaum and Rubin, 1983) and Mahalanobis distance (Mahalanobis, 1936; Rubin, 1980)—were chosen, and a greedy (i.e., nearest neighbor) algorithm was applied. For propensity score matching, a caliper of 0.2 was used (Austin, 2011). A *p*-value of less than 0.05 was considered statistically significant in all analyses. Paired data were analyzed using the McNemar test. Statistical analysis was performed using R software (R Core Team, 2019).

## Results

Between 1 March 2020, and 30 November 2020, 378,862 patients enrolled in Meuhedet HMO, including 255,355 (66.9%) aged 18 years and above, underwent SARS-CoV-2 PCR testing. Of these, 44,397 (17.52%) adults had at least one positive SARS-CoV-2 test. On average, patients who tested positive were younger (37.57 ± 16.37 vs. 41.40 ± 17.79 years), more likely to be males (55.9% vs. 46.7%), and less likely to smoke (7.8% vs. 15.2%) compared to those who tested negative. Among the adult cohort, 12,066 (4.7%) patients were prescribed PPIs 3 months before SARS-CoV-2 testing. Additional comparisons are presented in Table 1.

**TABLE 1:** Characteristics of patients who underwent PCR testing for SARS-CoV-2.

	SARS-CoV-2 negative	SARS-CoV-2 positive	*p*-value
n	208958	44397	
Age [mean (SD)]	41.40 (17.79)	37.57 (16.37)	<0.001
Male (%)	97633 (46.7)	24832 (55.9)	<0.001
BMI kg/m^2^ (mean(SD)	26.45 (5.48)	26.55 (5.86)	0.018
Smoking (%)	31769 (15.2)	3485 (7.8)	<0.001
Diabetes Mellitus (%)	16063 (7.7)	3047 (6.9)	<0.001
Ischemic Heart disease (%)	7676 (3.7)	1080 (2.4)	<0.001
Hypertension (%)	13852 (6.6)	2041 (4.6)	<0.001
Sector (%)			<0.001
Arab	35717 (17.1)	6832 (15.4)	
Jewish secular/orthodox	112016 (53.6)	13109 (29.5)	
Jewish ultra-orthodox	61225 (29.3)	24456 (55.1)	

BMI, body mass index.

**FIGURE 1:**
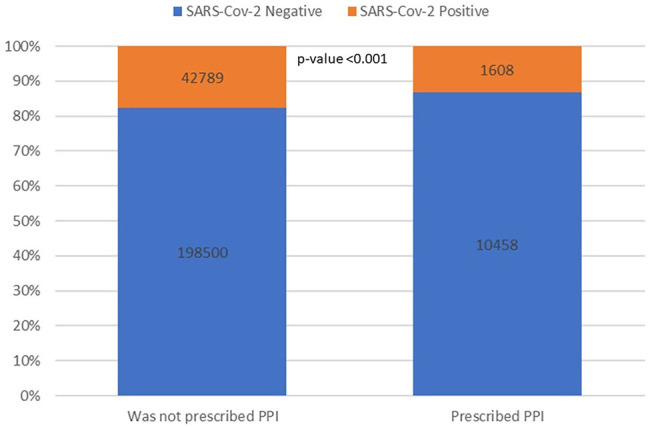
PPIs and SARS-CoV-2 testing results. The numbers on the bars represent the number of patients in each sub-category, whereas the inner partition into orange and blue represents the conditional distribution of SARS-CoV-2 infection.

Overall, those prescribed PPIs were less likely than others to have a positive SARS-CoV-2 test (17.7% vs. 13.3%, *p* value < 0.001) (Figure 1) in the crude analysis. However, in a multivariate logistic regression model controlling for age, gender, sector, smoking status, BMI, history of DM, COPD, IHD, hypertension, and prior FBG levels PPIs were not associated with SARS-CoV-2 infection (*p* value = 0.1 adjusted OR (aOR) 0.94, 95% CI—0.88–1.01) (Supplementary Table S1). When patients were matched by propensity score algorithm based on the above covariates (n = 6835 pairs of patients), PPIs were not associated with SARS-CoV-2 infection (*p* value = 0.06, aOR = 0.9 95% CI 0.82–1.0) (Supplementary Table S2).

**FIGURE 2:**
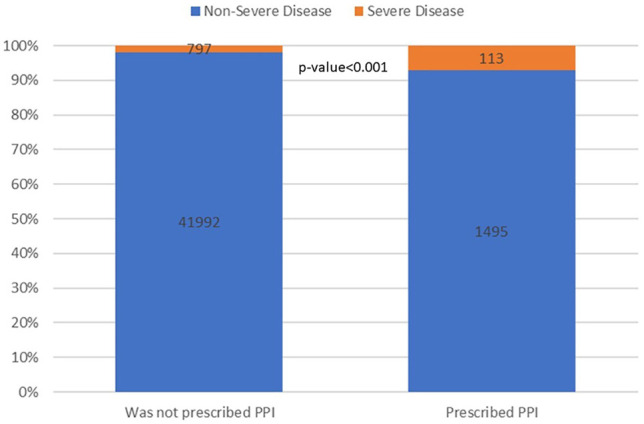
PPIs and COVID-19 severity. The numbers on the bars represent the number of patients in each sub-category, whereas the inner partition into orange and blue represents the conditional distribution of severity.

**FIGURE 3:**
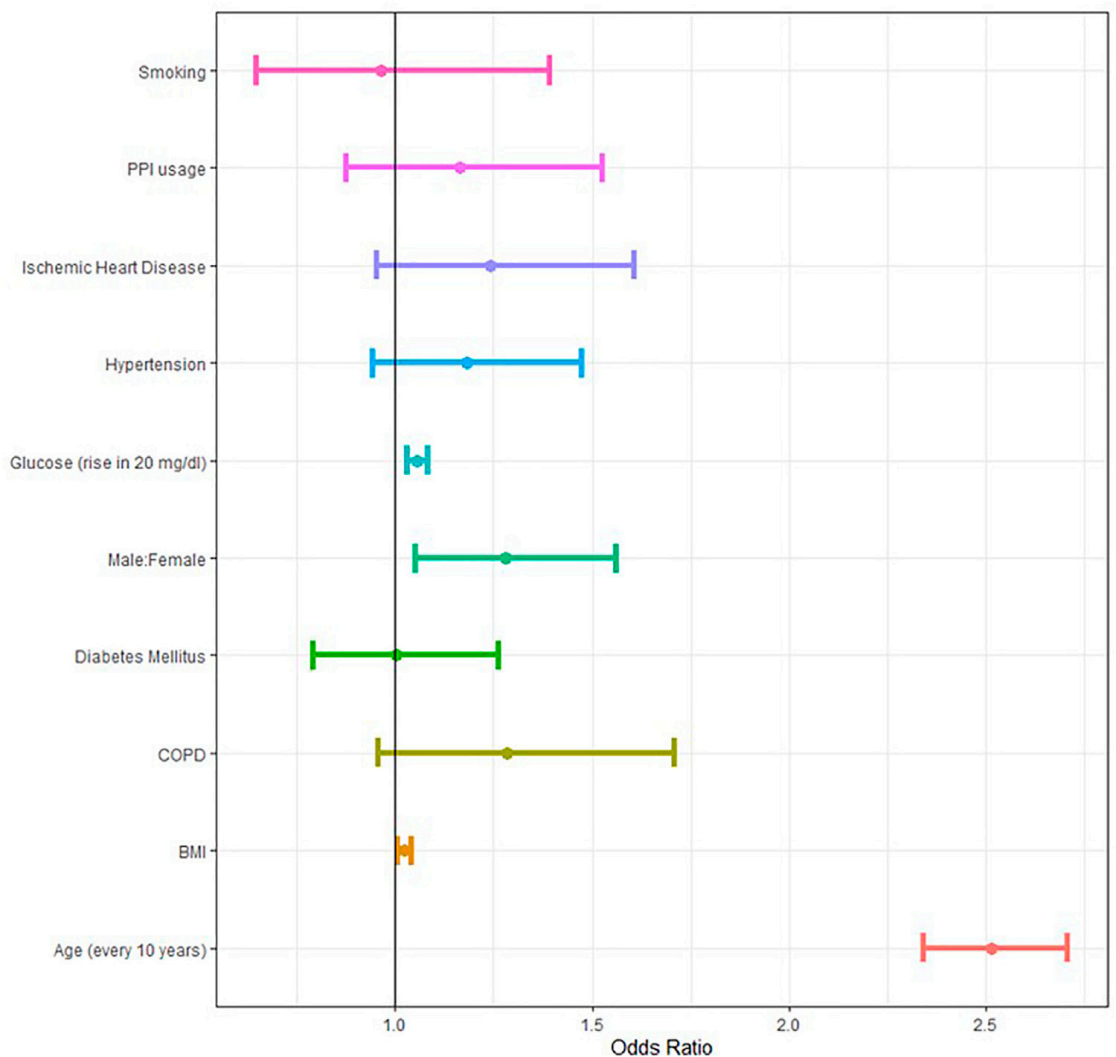
Forest plot of risk factors associated with severe COVID-19 disease (death/ICU/hospitalization longer than 10 days).

Among patients who tested positive for SARS-CoV-2, 910 (2%) had a severe course. Of these, 349 died, 253 were hospitalized in the ICU, and 642 were hospitalized for 10 days or more (non-mutually exclusive). On average, patients with severe infection were older (67.48 ± 18.49 vs. 28.75 years ± 18.07), more likely to be obese (BMI>30 kg/m^2^, 43.7% vs. 19.3%), more likely to have a diagnosis of DM (35.8% vs. 4.4%), and more likely to have a diagnosis of IHD (17.4% vs. 1.5%). Pre-COVID-19 FBG was higher among patients with severe COVID-19 (114.03 vs. 94.12 *p* value < 0.001). This finding was statistically significant when analyzed in a subset of patients without a documented diagnosis of DM (95.32 vs. 89.02 *p* value < 0.001) as well borderline-significant in patients diagnosed with DM (141.66 vs. 133.67 *p* value = 0.06). Gender and smoking were not found to be significantly associated with severe infection. Additional comparisons are shown in Table 2. Bivariate analysis showed that patients who were prescribed PPIs were more likely to suffer from severe infection (Figure 2) (12.4% vs. 3.4% *p*-value <0.001). Howver, in multivariate logistic regression controlling for age, gender, BMI, DM, history of smoking, hypertension, COPD, IHD, and FBG levels, there was no significant association between PPIs and severe COVID-19 (*p* value = 0.28, Figure 3) (Supplementary Table S3). In a similar multivariate logistic regression with substituting HbA1C for FBG, PPIs were not associated with severe disease (*p*-value = 0.32).

**TABLE 2:** Characteristics of patients with severe COVID-19, compared to those with non-severe COVID-19.

	Non-severe disease	Severe disease	*p*-value
n	63107	920	
Age [mean (SD)]	28.75 (18.07)	67.48 (18.49)	<0.001
Male (%)	35415 (56.1)	528 (57.4)	0.46
BMI kg/m^2^ [mean (SD)]	24.54 (6.4)	29.84 (6.17)	<0.001
Sector (%)			<0.001
Arab	8686 (13.8)	171 (18.6)	
Jewish secular/orthodox	16942 (26.8)	441 (47.9)	
Jewish Ultra-Orthodox	37479 (59.4)	308 (33.5)	
Diabetes Mellitus (%)	2756 (4.4)	329 (35.8)	<0.001
Ischemic Heart disease (%)	932 (1.5)	160 (17.4)	<0.001
Smoking (%)	3526 (5.6)	50 (5.4)	0.898
Hypertension (%)	1790 (2.8)	255 (27.7)	<0.001

When patients were matched by the Mahalanobis distance algorithm based on the above covariates (n = 655 pairs, Supplementary Table S4), PPIs were not associated with increased risk of severe COVID-19 (*p*-value = 0.48). In addition, a propensity score-matched cohort was constructed using the same parameters as mentioned in the logistic regression, and, in this analysis, (n = 1,019 pairs) PPIs were also not associated with an increased risk of severe COVID-19 (*p*-value = 0.25).

**FIGURE 4:**
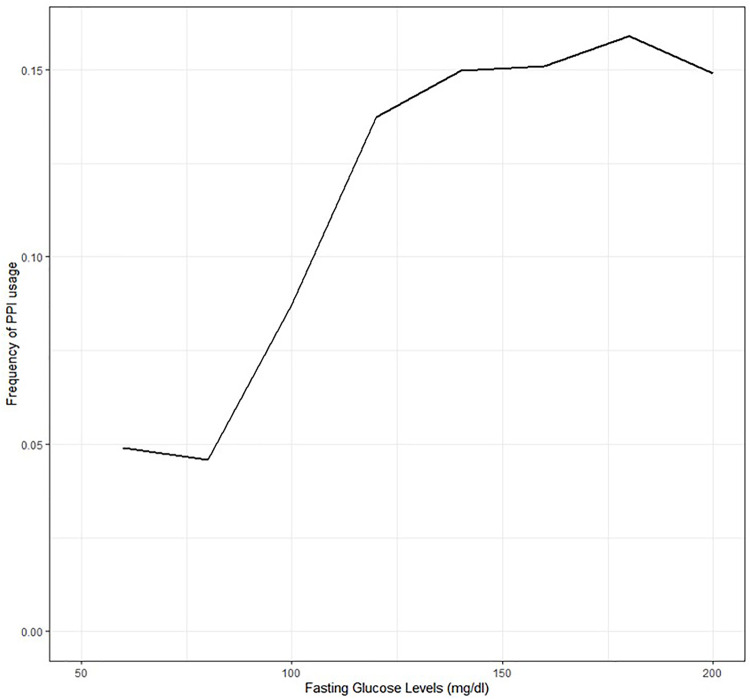
Percentage of PPI prescription given prior fasting glucose range.

To further study the impact of FBG as a confounder in the potential association of PPIs and severe COVID-19, we tested the association between FBG and PPI use. Higher FBG levels were positively associated with the use of PPIs (Figure 4). Furthermore, a multivariate logistic regression model controlling for age, gender, BMI>30 kg/m^2^, hypertension, and smoking, showed an association between higher fasting blood glucose levels and PPIs (p-value <0.001, Supplementary Table S5). A similar association was seen with HbA1c levels (p-value <0.001).

## Discussion

In this nationwide cohort of 255,355 adults who underwent SARS-CoV-2 testing, PPI exposure (based on the prescription of PPIs) was not associated with SARS-CoV-2 positivity after controlling for comorbidities and sociodemographic factors. Furthermore, among the 44,397 patients who tested positive for SARS-CoV-2, PPIs were not associated with severe COVID-19, defined as death, ICU stay, or hospitalization of 10 days or longer. As with prior studies assessing the potential adverse events of PPIs in a retrospective cohort and non-randomized studies, confounders likely affect the hypothesized impact of PPIs on diseases and outcomes (Almario et al., 2020; Farsalinos et al., 2020; Hariyanto et al., 2020; Lee et al., 2020; Li et al., 2020; Ramachandran et al., 2020; Fan et al., 2021; Luxenburger et al., 2021).

Similar to other studies, our analysis showed that advanced age, male gender, and elevated BMI were associated with severe COVID-19 (Deng and Peng, 2020; Zhou et al., 2020; Zhu et al., 2020; Shauly-Aharonov et al., 2021). Since the data in this study was from a large HMO, the analysis could control for confounders found in serum blood tests and not simply rely on diagnoses lists. This is vital as patients with DM and SARS-CoV-2 who had poorly controlled blood glucose levels had significantly worse outcomes (Singh and Singh, 2020; Zhu et al., 2020). In addition, impaired fasting glucose, even without a personal history of DM, was associated with more severe COVID-19 outcomes (Zhang et al., 2020). In this analysis both a diagnosis of DM and FBG levels were included as potential confounders for the regression model. While both confounders are associated with blood sugar levels, it is imperative to differentiate between patients who receive glucose-lowering agents and those who do not. As FBG is affected by the hours that the patient fasted, an additional regression was constructed using HbA1c instead of FBG showing similar results.

The association of PPIs and increased FBG levels can be explained in several directions. PPIs may serve as a risk factor for DM. Yuan et al. analyzed three prospective studies and found a 24% increased risk of developing DM among patients who received PPIs than those who did not (Lee et al., 2021). On the other hand, GI symptoms and disorders are common in patients with DM, and some are correlated with increased glucose levels (Rayner et al., 2000; Careyva and Stello, 2016; Du et al., 2018). Patients with DM are more likely to receive PPIs (Zawada et al., 2018). As such, adjusting for both a history of DM and poor glucose control is vital when assessing PPIs’ potential adverse effects.

This study is a historical cohort and is limited, similar to other administrative database studies (Etminan et al., 2020). A prospective study would be the ideal way to assess PPIs and SARS-CoV-2. However, the feasibility of performing such a study, particularly during the pandemic, would be extremely difficult (Emani et al., 2021). A major limitation of historical cohort studies assessing the association between prescribed pharmaceuticals and outcomes is confounding by indication (Schneeweiss and Avorn, 2005). The underlying disease or complaint for which PPIs were prescribed could not be discerned, and these unknown indications may be related to COVID-19 outcomes. In addition, PPI dosage or length of PPI use could not be assessed. Nonetheless, the PPI prescribing data in our cohort is objective pharmacy data and not based on self-reporting. Additional confounders that impact both FBG and COVID-19 outcomes may have been missing and not controlled for during statistical analysis (Martins, 2015).

During the initial months of the COVID-19 pandemic, many people refused to be tested for SARS-CoV-2 out of fear of the isolation restrictions imposed on those who tested positive. Thus, SARS-CoV-2 positive patients were not compared to those who were not tested for SARS-CoV-2. Untested individuals may have been infected with SARS-CoV-2 and not presented for testing.

An additional limitation is that patients may have taken PPIs without prescription thus misclassifying the exposure and biasing toward the null, this comparison can underestimate the number of COVID-19 patients due to false-negative results. The retrospective nature of the data also limited the definition of severe disease. Hospital data only included length of hospitalization and need for ICU, and we did not have access to in-hospital metrics such as the need for supplemental oxygen. The financial compensation that the HMO pays to the hospital is dependant on hospitalization length and ICU stay; therefore these are validated and reliable data. A composite endpoint of severe disease including death, ICU, and prolonged hospitalization was used. As there is universal health care coverage in Israel, a prolonged hospitalization will not be related to insurance bureaucracy and is a surrogate for more severe disease. Furthermore, effective interventions in COVID-19 are associated with shorter hospitalizations and a milder course of disease (RECOVERY Collaborative Group, 2020). Finally, the degree of physical activity, anti-diabetic/anti-hypertensive medication usage, dietary intake, and use of nutritional supplements are potential confounders when assessing FBG and COVID-19 outcome. However, this data was not available due to this study’s retrospective administrative database manner.

Nonetheless, this study has significant strengths, including a large number of patients and the inclusion of blood test results to assess and control for confounders. It is noteworthy that a strong and statistically significant crude association was found which was obliterated using multiple techniques to control confounding including multivariable analysis and propensity score matching. While other studies may have seen an association between PPIs and SARS-CoV-2 infectivity or death, this may have been a result of protopathic bias, or outcomes being attributed to treatments as opposed to the conditions being treated as was recently demonstrated in a study of 1.9 million patients showing no increased mortality risk with PPI use (Baik et al., 2021). While these findings may provide reassurance that pre-infection PPI exposure is not associated with worse SARS-CoV-2 prognosis, it must be emphasized that PPIs may be overutilized, and use should be limited to appropriate indications and for appropriate durations (Moayyedi, 2020).

In conclusion, after controlling for confounders, this large retrospective cohort study of 255,355 adults shows no association between PPIs and SARS-CoV-2 PCR positivity and severity. Our results support continued use of PPIs for patients with an appropriate indication without concern for increased risk of COVID-19.

## What is Known

• Proton pump inhibitors (PPI) are commonly used
• Prior studies evaluating the association of PPI use with Coronavirus disease 2019 (COVID-19) severity showed conflicting results.

## What is New Here

• Elevated fasting blood glucose is associated with both severe COVID-19 and PPI use.
• When controlling for fasting blood glucose, no significant association was found between PPIs and COVID-19 severity.

## Data Availability

The datasets presented in this article are not readily available because there are ethical restrictions on sharing our data set because data contain potentially identifying patient information. These restrictions were imposed by the ethics committee of Meuhedet HMO who own the data. Requests to access the datasets should be directed to Liron Yitzchaki, coordinator of Meuhedet research center liron.y3@meuhedet.co.il.
